# DHU-Pred: accurate prediction of dihydrouridine sites using position and composition variant features on diverse classifiers

**DOI:** 10.7717/peerj.14104

**Published:** 2022-10-27

**Authors:** Muhammad Taseer Suleman, Tamim Alkhalifah, Fahad Alturise, Yaser Daanial Khan

**Affiliations:** 1 Department of Computer Science, School of Systems and Technology, University of Management & Technology, Lahore, Pakistan; 2 Department of Computer, College of Science and Arts in Ar Rass Qassim University, Ar Rass, Qassim, Saudi Arabia

**Keywords:** Prediction, Dihydrouridine, Uridine modifications, Machine learning, Statistical moments, Classification, Random Forest, DHU-Pred, Post Transcriptional Modification, RNA

## Abstract

**Background:**

Dihydrouridine (D) is a modified transfer RNA post-transcriptional modification (PTM) that occurs abundantly in bacteria, eukaryotes, and archaea. The D modification assists in the stability and conformational flexibility of tRNA. The D modification is also responsible for pulmonary carcinogenesis in humans.

**Objective:**

For the detection of D sites, mass spectrometry and site-directed mutagenesis have been developed. However, both are labor-intensive and time-consuming methods. The availability of sequence data has provided the opportunity to build computational models for enhancing the identification of D sites. Based on the sequence data, the DHU-Pred model was proposed in this study to find possible D sites.

**Methodology:**

The model was built by employing comprehensive machine learning and feature extraction approaches. It was then validated using in-demand evaluation metrics and rigorous experimentation and testing approaches.

**Results:**

The DHU-Pred revealed an accuracy score of 96.9%, which was considerably higher compared to the existing D site predictors.

**Availability and Implementation:**

A user-friendly web server for the proposed model was also developed and is freely available for the researchers.

## Introduction

**Figure 1:**
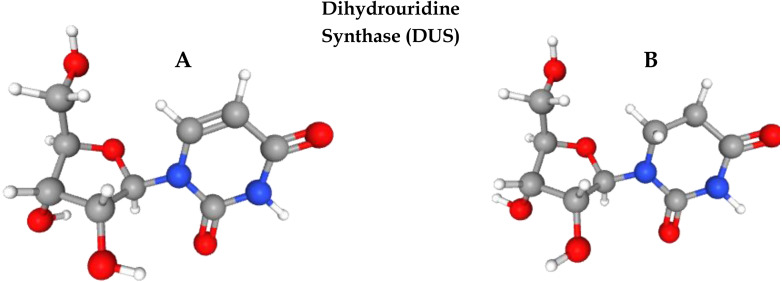
Formation of dihydrouridine from uridine. (A) 3D chemical structure of uridine. (B) Formation of Dihydrouridine (3D structure) through Dihydrouridine synthase (DUS) enzyme.

Post-transcriptional modification (PTM) is the process of chemical alteration of primary ribonucleic acid (RNA) to produce a mature RNA that helps in performing different cell functions (El Allali, Elhamraoui & Daoud, 2021). So far, more than 150 PTMs have been identified in RNA (Boccaletto et al., 2018). Uridine is a primary nucleoside that is composed of uracil and ribose. Several enzymes play a pivotal role in uridine modification. Among these modifications, dihydrouridine (D) and pseudouridine (Y) are the most prevalent modifications due to their roles in transfer RNA (tRNA) folding, gene expression, codon binding, and structural flexibility of tRNA. Eukaryotes, bacteria, and archaea all have high levels of this modification. Dihydrouridine base is formed at the uridine base by reducing the carbon–carbon double bond at positions 5 and 6. D formation is catalysed by an enzyme known as dihydrouridine synthase (Dus), from the flavin enzyme family, occurring in prokaryotes in three forms known as DusA, DusB, and DusC. It has been observed that D modification in human tRNA can be the cause of pulmonary carcinogenesis (Tseng, Medina & Randerath, 1978; Kato et al., 2005). Figure 1 shows the three-dimensional chemical structure of uridine and dihydrouridine.

The D modification is non-planar due to the lack of a double bond, which prevents base stacking. The structural flexibility, conformational folding, and stability of the tRNA structure are all strengthened by this modification (Dyubankova et al., 2015). The D site prediction is critical for fully comprehending its potential functions. Site-directed mutagenesis and mass spectrometry have been proposed as methods for detecting D modifications, although both are complex and time-consuming (Madec et al., 2003). The availability of sequence-based datasets has increased the possibility of applying computational intelligence methods for the prediction of PTM sites.

Researchers predicted the uridine modifications in the tRNA sequence through a support vector machine (SVM) (Panwar & Raghava, 2014). A three-stage approach was used in their research, including training and validation of new tRNA sequences on the previous model and specie-wise dataset training and validation. Liu, Chen & Lin (2020) proposed a predictor, XG-PseU, for the identification of pseudo uridine modification through an optimal feature selection method. Feng et al. (2019) proposed a method for the detection of D modification in *Saccharomyces cerevisiae* using an ensemble classifier. Three different feature extraction approaches were used by the authors, including nucleotide physicochemical property (NPCP), pseudo dinucleotide composition (PseDNC), and secondary structure component (SSC) with SVM for classification. For comparison, an SVM-based ensemble approach was adopted based on voting among these three extraction features. However, the metrics results were not optimal, revealing 83.08% accuracy (*Acc*), 89.71% specificity (*S*_*p*_), 76.47% sensitivity (*S*_*n*_), and a 0.62 Matthews correlation coefficient (*MCC*). Similarly, Xu et al. (2019) developed a predictor, iRNAD, for the prediction of D modification based on RNA samples of five species. The samples were encoded using nucleotide chemical property (NCP) and nucleotide density. The SVM was utilised as a classification model, and the jackknife test was used to assess the model’s performance. The proposed model outlined a 96.18% *Acc* with *S*_*p*_ and *S*_*n*_ scores of 98.13% and 92.05%, respectively. Dou et al. (2021) published recently in which they proposed a model, iRNAD-XGBoost, in consideration of the imbalance problem using a hybrid sampling method and the feature selection method. However, an independent test of the model revealed the values of *Acc*, *S*_*n*_, *S*_*p*_, and *MCC* to be 93.75%, 91.67%, 94.74%, and 0.86, respectively.

The current study focused on the prediction of D sites in tRNA using a novel method for feature extraction from the RNA sequences obtained from three species, including *Homosapiens*, *Mus musculus*, and *Saccharomyces cerevisiae*. A novel methodology was adapted for the extraction and representation of feature vectors based on the position as well as the composition of nucleotide bases through the incorporation of statistical moments to increase the prediction capability of the model (Amanat et al., 2019; Naseer et al., 2020; Barukab et al., 2019). The development and training of computationally intelligent models was aided by these feature vectors. The performance of all models was assessed through various testing methods such as the independent set test, jackknife test, and k-fold cross-validation. The overall accuracy of the model was assessed through *S*_*n*_, *S*_*p*_, *MCC*, and *Acc.*

**Figure 2:**
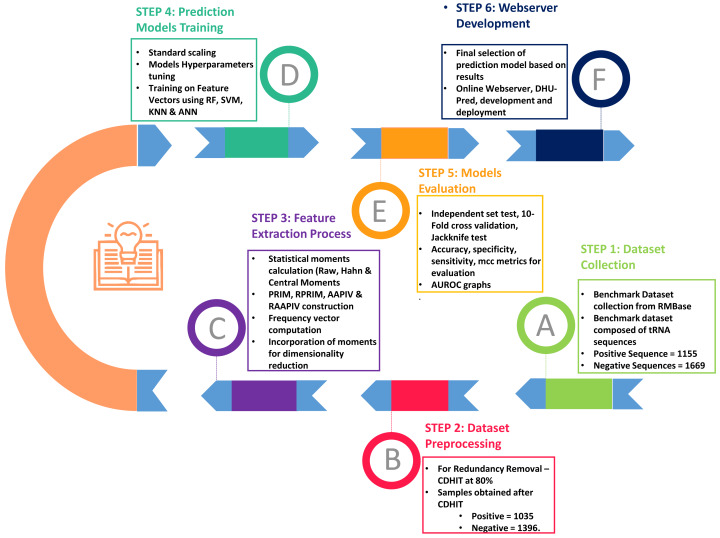
Flow chart of the methodology.

As shown in Fig. 2, the entire approach employed in this work included dataset collection, sample formulation, prediction model training, and model evaluation. Finally, DHU-Pred, a publicly accessible web server, was created to aid D modification research.

## Materials & Methods

### Dataset collection

The collection of the benchmark dataset was the initial phase of the research. The tRNA sequences were considered for the feature extraction and prediction model training in the current investigation. The sequences were obtained from RMBase (Xuan et al., 2017), also used by Xu et al. (2019), Feng et al. (2019), and Dou et al. (2021). The benchmark dataset contained data from three species, including *Homosapiens* (Human), *Mus musculus* (Mouse), and *Saccharomyces cerevisiae* (Yeast) related to D modification.

#### Positive and negative samples

Each data set sample was composed of 41 nucleotides with *U* at its center, *i.e.,* at position 21. The experimental results revealed the optimal accuracy scores were achieved using a sequence length of 41 nucleotides. In addition, an RNA sample containing the D site was expressed as mentioned in Eq. (1).

(1) \begin{eqnarray*}P \left( U \right) ={P}_{-\epsilon }{P}_{- \left( \epsilon -1 \right) }....{P}_{-2}{P}_{-1}U{P}_{+1}{P}_{+2}....{P}_{+ \left( \epsilon -1 \right) }{P}_{+\epsilon }.\end{eqnarray*}

In Eq. (1), the symbol *U* represents uridine (U), the center of nucleotide sequences, and the subscript value *ϵ* is set as 20. Thus, the total length of the nucleotide sequence is (2 *ϵ*+1). *P*_−*ϵ*_ represents the *ϵ*-th upstream nucleotide from the central uridine and *P*_+*ϵ*_ represents the *ϵ*-th downstream nucleotide. The positive samples signify the sequence with D modification, whereas the negative samples express the sequences without D modification. The total positive and negative sites of all three previously mentioned species were 1,155 and 1,669, respectively. However, removing redundant samples with CD-HIT (16) at 0.80 reduced the sample size to 1,035 positive and 1,396 negative samples.

#### Sequence logo

**Figure 3:**
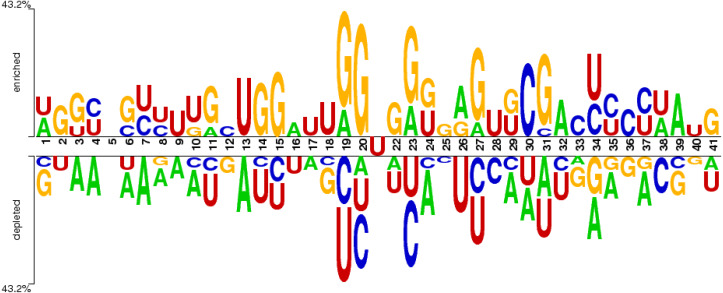
Distribution of nucleotides in the sample dataset with U in the middle.

The distribution of nucleotide bases in the obtained sequences can be illustrated with the help of the sequence logo. An online Two Sample Logo tool (Vacic, Iakoucheva & Radivojac, 2006) was used for the said purpose. The sequence logo shown in Fig. 3 expressed the distribution of cytosine (C), guanine (G), adenine (A), and uracil (U) in the dataset.

The nucleotide base distribution from the centre nucleotide base (*i.e.,* uracil) is different between positive sites (from position 22 to 41) and negative sites (from position 1 to 20). It can be observed from Fig. 3 that G and C are enriched in the region located from position 19 (negative site) to position 31 (positive site). However, the base A is symmetrically distributed along the whole region within all nucleotides. The nucleotide base U is mostly concentrated in and around the centre of all RNA samples.

### Feature generation and representation from RNA samples

Encoding RNA sequences into feature vectors is one of the most prevalent steps because computational models cannot handle and process biological sequences directly. As a result, statistical analysis of the acquired samples can better retrieve the obscured information within the sequences. The current study dealt with the feature generation mechanism based on the position and composition of nucleotides within a given sequence. Chou suggested the pseudo amino acid composition (PseAAC) as one of the most popular and effective ways of dealing with the problem of sequence pattern loss. The current study implemented a similar approach to PseAAC in pseudo K-tuple nucleotide composition for feature vector generation (Chen, Chou & Chen, 2015; Xiao et al., 2019; Awazu, 2017). These vectors served as input for model training, as mentioned in these comprehensive research works Mahmood et al. (2020) and Shah & Khan (2020). For the current research, the feature vectors were developed based on the position and composition of nucleotides in each sequence. The samples in the dataset were characterised as follows using the nucleotide formulation, ${P}_{\epsilon } \left( K \right) $,described in Eq. (2).

(2) \begin{eqnarray*}{P}_{\epsilon } \left( K \right) ={ \left[ {\text{\pounds}}_{1}{\text{\pounds}}_{2}{\text{\pounds}}_{3}\ldots {\text{\pounds}}_{U}\ldots {\text{\pounds}}_{\Omega } \right] }^{T}.\end{eqnarray*}

Where, at K-tuple nucleotide, £ represents each component in a vector based on the feature generation mechanism adopted in this research. Where *T* represents the transpose of the accumulated feature formulation. Each nucleotide sample of a specific site was 41 base pairs (bp) in length and is expressed in Eq. (3).

(3) \begin{eqnarray*}P={R}_{1}{R}_{2}{R}_{3}\cdots {R}_{18}{R}_{19}{\mathbi{R}}_{21}\cdots {R}_{39}{R}_{40}{R}_{41}.\end{eqnarray*}

In Eq. (3), ***R***_21_ = ***U*** and *R*_1_ (*n* = 1, 2………, 41; n ≠ 21) represent any nucleotide such as cytosine, guanine, adenine, and uracil.

#### Statistical moment calculation

The quantification of the collected nucleotide sequences is based on their composition and position. Moments have been applied to various data distributions by statisticians and data analysts (Malebary & Khan, 2021). For this purpose, central, raw, and Hahn moments were used in the feature extraction process. Raw and Hahn moments are scale and location variant, whereas central moments are scale and vicinity variant. Therefore, the dataset’s mean, asymmetry, and variance were calculated using raw and central moments. On the contrary, Hahn moments were calculated by the reference of Hahn polynomials to maintain the sequence order information (Khan et al., 2020). Butt et al. (2016) used these moments as a means for feature extraction, which was used to identify membrane proteins. A matrix *K*′ in Eq. (4) is a m*n two-dimensional matrix in which a single element, *k*_*mn*_, represents the *n*th nucleotide base in *m*th sequence.

(4) \begin{eqnarray*}{K}^{{^{\prime}}}= \left[ \begin{array}{@{}cccc@{}} \displaystyle {k}_{11}&\displaystyle {k}_{12}&\displaystyle \ldots &\displaystyle {k}_{1n}\\ \displaystyle {k}_{21}&\displaystyle {k}_{22}&\displaystyle \ldots &\displaystyle {k}_{2n}\\ \displaystyle \vdots &\displaystyle \vdots &\displaystyle \ddots &\displaystyle \vdots \\ \displaystyle {k}_{m1}&\displaystyle {k}_{m2}&\displaystyle \ldots &\displaystyle {k}_{mn} \end{array} \right] .\end{eqnarray*}

Raw moments were used to extract location variant features by calculating the dataset’s mean, variance, and unequal probability distribution. Raw moments expressed in Eq. (5) where, *u* + *v* is the sum of raw moments and *R*_00_, *R*_01_, *R*_10_, *R*_11_, *R*_12_, *R*_21_, *R*_30_, *R*_03_, were calculated up to 3rd-degree polynomial.

(5) \begin{eqnarray*}{R}_{uv}=\sum _{a=1}^{m}\sum _{b=1}^{m}{a}^{u}{b}^{v}{\beta }_{ab}.\end{eqnarray*}

Central moments do not depend upon the location. Instead, these are related to the composition and shape of the distribution (TLo & Don, 1989). The central moments were calculated based on the deviations of the random variable from the mean. For this study, the central moments were computed as expressed in Eq. (6).

(6) \begin{eqnarray*}{n}_{ij}=\sum _{b=1}^{n}\sum _{q=1}^{n}{ \left( b-x \right) }^{i}{ \left( q-y \right) }^{j}{\beta }_{bq}.\end{eqnarray*}

Hahn moments were computed using Hahn polynomials. Hahn moments calculation mentioned in the Eq. (7).

(7) \begin{eqnarray*}{h}_{n}^{u,v} \left( r,N \right) ={ \left( N+V-1 \right) }_{n}{ \left( N-1 \right) }_{n}\times \sum _{k=0}^{n}{ \left( -1 \right) }^{k} \frac{{ \left( -n \right) }_{k}{ \left( -r \right) }_{k}{ \left( 2N+u+v-n-1 \right) }_{k}}{{ \left( N+v-1 \right) }_{k}{ \left( N-1 \right) }_{k}} \frac{1}{k{!}} .\end{eqnarray*}

The following expression Eq. (8) was used to determine the orthogonal normalized Hahn of the two-dimensional data.

(8) \begin{eqnarray*}{H}_{ij}=\sum _{q=0}^{N-1}\sum _{p=0}^{N-1}{\beta }_{ij}{h}_{j}^{\widetilde {u,v}} \left( q,N \right) {h}_{j}^{\widetilde {u,v}} \left( p,N \right) , m,n=0,1,\ldots ,N-1.\end{eqnarray*}

#### Construction of Position Relative Incidence Matrix (PRIM)

The current study focused on improving the model’s prediction abilities. Therefore, a complete feature generation model was required for the said purpose. The relative positions of nucleotides within an RNA sequence are helpful and become the basis for mathematical formulation. For this purpose, three types of position relative incidence matrix (PRIM) were constructed by considering single nucleotide composition (SNC), di-nucleotide composition (DNC), and tri-nucleotide composition (TNC). These matrices were created to reveal the relative positions of nucleotide bases, which helped in comprehensively quantizing the relative positions of nucleotides. The matrix, *A*_*Prim*_, is a 4*4 matrix Eq. (9) that produced a total of 16 coefficients.

(9) \begin{eqnarray*}{A}_{prim}= \left[ \begin{array}{@{}cccc@{}} \displaystyle {{null}}_{A\rightarrow A}&\displaystyle {{null}}_{A\rightarrow G}&\displaystyle {{null}}_{A\rightarrow U}&\displaystyle {{null}}_{A\rightarrow C}\\ \displaystyle {{null}}_{G\rightarrow A}&\displaystyle {{null}}_{G\rightarrow G}&\displaystyle {{null}}_{G\rightarrow U}&\displaystyle {{null}}_{G\rightarrow C}\\ \displaystyle {{null}}_{U\rightarrow A}&\displaystyle {{null}}_{U\rightarrow G}&\displaystyle {{null}}_{U\rightarrow U}&\displaystyle {{null}}_{U\rightarrow C}\\ \displaystyle {{null}}_{C\rightarrow A}&\displaystyle {{null}}_{C\rightarrow G}&\displaystyle {{null}}_{C\rightarrow U}&\displaystyle {{null}}_{C\rightarrow C} \end{array} \right] .\end{eqnarray*}

Where, Ԏ _*i*→*j*_, represents the relative position of any nucleotide (*i.e.,* A, C, U, or G) to other nucleotides. The matrix, *B*_*Prim*_, is a 16*16 matrix Eq. (10) that denotes the DNC producing 16 unique combinations of nucleotides (*i.e.,* AA, AG, AU, …, CG, *CU, CC*). This matrix yielded a total of 256 coefficients. However, with the fusion of statistical moments, only 30 coefficients were derived.

(10) \begin{eqnarray*}{B}_{prim}= \left[ \begin{array}{@{}ccccccc@{}} \displaystyle {{null}}_{AA\rightarrow AA}&\displaystyle {{null}}_{AA\rightarrow AG}&\displaystyle {{null}}_{AA\rightarrow AU}&\displaystyle \ldots &\displaystyle {{null}}_{AA\rightarrow j}&\displaystyle \ldots &\displaystyle {{null}}_{AA\rightarrow CC}\\ \displaystyle {{null}}_{AG\rightarrow AA}&\displaystyle {{null}}_{AG\rightarrow AG}&\displaystyle {{null}}_{AG\rightarrow AU}&\displaystyle \ldots &\displaystyle {{null}}_{AG\rightarrow j}&\displaystyle \ldots &\displaystyle {{null}}_{AG\rightarrow CC}\\ \displaystyle {{null}}_{AU\rightarrow AA}&\displaystyle {{null}}_{AU\rightarrow AG}&\displaystyle {{null}}_{AU\rightarrow AU}&\displaystyle \ldots &\displaystyle {{null}}_{AU\rightarrow j}&\displaystyle \ldots &\displaystyle {{null}}_{AU\rightarrow CC}\\ \displaystyle \vdots &\displaystyle \vdots &\displaystyle \vdots &\displaystyle &\displaystyle \vdots &\displaystyle \vdots &\displaystyle \vdots \\ \displaystyle {{null}}_{GA\rightarrow AA}&\displaystyle {{null}}_{GA\rightarrow AG}&\displaystyle {{null}}_{GA\rightarrow AU}&\displaystyle \ldots &\displaystyle {{null}}_{GA\rightarrow j}&\displaystyle \ldots &\displaystyle {{null}}_{GA\rightarrow CC}\\ \displaystyle \vdots &\displaystyle \vdots &\displaystyle \vdots &\displaystyle &\displaystyle \vdots &\displaystyle \vdots &\displaystyle \vdots \\ \displaystyle {{null}}_{N\rightarrow AA}&\displaystyle {{null}}_{N\rightarrow AG}&\displaystyle {{null}}_{N\rightarrow AU}&\displaystyle \ldots &\displaystyle {{null}}_{N\rightarrow j}&\displaystyle \ldots &\displaystyle {{null}}_{N\rightarrow CC} \end{array} \right] .\end{eqnarray*}

The matrix, *C*_*Prim*_, is a 64*64 matrix Eq. (11) representing 64 unique tri-nucleotide combinations (*i.e.,* AAA, AAG, AAU, …., CCG, CCU, CCC). *C*_*Prim*_ yielded 4,096 coefficients. However, with the incorporation of central, raw, and Hahn moments, 30 coefficients were computed.

(11) \begin{eqnarray*}{C}_{prim}= \left[ \begin{array}{@{}ccccccc@{}} \displaystyle {\Psi }_{AAA\rightarrow AAA}&\displaystyle {\Psi }_{AAA\rightarrow AAG}&\displaystyle {\Psi }_{AAA\rightarrow AAU}&\displaystyle \ldots &\displaystyle {\Psi }_{AAA\rightarrow j}&\displaystyle \ldots &\displaystyle {\Psi }_{AAA\rightarrow CCC}\\ \displaystyle {\Psi }_{AAG\rightarrow AAA}&\displaystyle {\Psi }_{AAG\rightarrow AAG}&\displaystyle {\Psi }_{AAG\rightarrow AAU}&\displaystyle \ldots &\displaystyle {\Psi }_{AAG\rightarrow j}&\displaystyle \ldots &\displaystyle {\Psi }_{AAG\rightarrow CCC}\\ \displaystyle {\Psi }_{AAU\rightarrow AAA}&\displaystyle {\Psi }_{AAU\rightarrow AAG}&\displaystyle {\Psi }_{AAU\rightarrow AAU}&\displaystyle \ldots &\displaystyle {\Psi }_{AAU\rightarrow j}&\displaystyle \ldots &\displaystyle {\Psi }_{AAU\rightarrow CCC}\\ \displaystyle \vdots &\displaystyle \vdots &\displaystyle \vdots &\displaystyle &\displaystyle \vdots &\displaystyle &\displaystyle \vdots \\ \displaystyle {\Psi }_{AAC\rightarrow AAA}&\displaystyle {\Psi }_{AAC\rightarrow AAG}&\displaystyle {\Psi }_{AAC\rightarrow AAU}&\displaystyle \ldots &\displaystyle {\Psi }_{AAC\rightarrow j}&\displaystyle \ldots &\displaystyle {\Psi }_{AAC\rightarrow CCC}\\ \displaystyle \vdots &\displaystyle \vdots &\displaystyle \vdots &\displaystyle &\displaystyle \vdots &\displaystyle &\displaystyle \vdots \\ \displaystyle {\Psi }_{N\rightarrow AAA}&\displaystyle {\Psi }_{N\rightarrow AAG}&\displaystyle {\Psi }_{N\rightarrow AAU}&\displaystyle \ldots &\displaystyle {\Psi }_{N\rightarrow j}&\displaystyle \ldots &\displaystyle {\Psi }_{N\rightarrow CCC} \end{array} \right] .\end{eqnarray*}

#### Reverse Position Relative Indices Matrix (RPRIM)

The main objective in feature vector determination is to extract as much information as possible to develop a reliable predictive model. Reversing the sequence order to get more embedded information within the sequences yielded a reverse position relative indices matrix (RPRIM). Eq. (12) states, *V*_*RPRIM*_, in which any arbitrary element, *R*_*i*→*j*_, represents the relative position value of the *i* th nucleotide base to the *j* th nucleotide. The calculation of RPRIM was carried out using mononucleotide, di-nucleotide, and tri-nucleotide combinations like PRIM matrices.

(12) \begin{eqnarray*}{V}_{RPRIM}= \left[ \begin{array}{@{}ccccccc@{}} \displaystyle {V}_{1\rightarrow 1}&\displaystyle {V}_{1\rightarrow 2}&\displaystyle {V}_{1\rightarrow 3}&\displaystyle \ldots &\displaystyle {V}_{1\rightarrow y}&\displaystyle \ldots &\displaystyle {V}_{1\rightarrow j}\\ \displaystyle {V}_{2\rightarrow 1}&\displaystyle {V}_{2\rightarrow 2}&\displaystyle {V}_{2\rightarrow 3}&\displaystyle \ldots &\displaystyle {V}_{2\rightarrow y}&\displaystyle \ldots &\displaystyle {V}_{2\rightarrow j}\\ \displaystyle {V}_{3\rightarrow 1}&\displaystyle {V}_{3\rightarrow 2}&\displaystyle {V}_{3\rightarrow 3}&\displaystyle \ldots &\displaystyle {V}_{3\rightarrow y}&\displaystyle \ldots &\displaystyle {V}_{3\rightarrow j}\\ \displaystyle \vdots &\displaystyle \vdots &\displaystyle \vdots &\displaystyle &\displaystyle \vdots &\displaystyle &\displaystyle \vdots \\ \displaystyle {V}_{x\rightarrow 1}&\displaystyle {V}_{x\rightarrow 2}&\displaystyle {V}_{x\rightarrow 3}&\displaystyle \ldots &\displaystyle {V}_{x\rightarrow y}&\displaystyle \ldots &\displaystyle {V}_{4\rightarrow j}\\ \displaystyle \vdots &\displaystyle \vdots &\displaystyle \vdots &\displaystyle &\displaystyle \vdots &\displaystyle &\displaystyle \vdots \\ \displaystyle {V}_{N\rightarrow 1}&\displaystyle {V}_{N\rightarrow 2}&\displaystyle {V}_{N\rightarrow 3}&\displaystyle \ldots &\displaystyle {V}_{N\rightarrow y}&\displaystyle \ldots &\displaystyle {V}_{N\rightarrow j} \end{array} \right] .\end{eqnarray*}

#### Frequency Matrices (FMs) generation

It is necessary to extract information about the location as well as the composition of the sequence for generating attributes. The frequency vector Ġ in Eq. (13), on the other hand, provided the count for each nucleotide in the sequence.

(13) \begin{eqnarray*}\text{\.{G}}= \left\{ {\delta }_{1},{\delta }_{2},\ldots ..,{\delta }_{n} \right\} .\end{eqnarray*}

Where *δ*_*i*_ represents the count of each *i*th nucleotide within a sequence. The frequency vector Ġ was computed for single as well as paired nucleotides.

#### Generation of Accumulative Absolute Position Incidence Vector (AAPIV)

The extraction of compositional information did not provide enough information regarding the position-specific calculation of each nucleotide. For this purpose, accumulative absolute position incidence vectors (AAPIVs) of lengths 4, 16, and 64 were computed, which are represented as *K*_*AAPIV*4_, *K*_*AAPIV*16_, and *K*_*AAPIV*64_ in Eqs. (14), (15) and (16) respectively.

(14) \begin{eqnarray*}{K}_{AAPIV4}& = \left\{ {\rho }_{1},{\rho }_{2},{\rho }_{3},{\rho }_{4}, \right\} \end{eqnarray*}

(15) \begin{eqnarray*}{K}_{AAPIV16}& = \left\{ {\rho }_{1},{\rho }_{2},{\rho }_{3},...,{\rho }_{15},{\rho }_{16}, \right\} \end{eqnarray*}

(16) \begin{eqnarray*}{K}_{AAPIV64}& = \left\{ {\rho }_{1},{\rho }_{2},{\rho }_{3},...,{\rho }_{63},{\rho }_{64} \right\} .\end{eqnarray*}

Any element *ρ*_*i*_ is computed as follows:

(17) \begin{eqnarray*}{\rho }_{i}={\mathop{\sum \nolimits }\nolimits }_{k=1}^{n}{p}_{k}.\end{eqnarray*}

#### Reverse Accumulative Absolute Position Incidence Vector (RAAPIV) generation

The reverse accumulative absolute position incidence vector (RAAPIV) helped explore hidden information related to the relative positions of nucleotides in the sequence. The length of RAAPIV was 4, 16, and 64, expressed as *K*_*RAAPIV*4_ Eq. (18), *K*_*RAAPIV*16_ Eq. (19), and *K*_*RAAPIV*64_ Eq. (20), respectively, but with reverse sequence order.

(18) \begin{eqnarray*}{K}_{RAAPIV4}& = \left\{ {\tau }_{1},{\tau }_{2},{\tau }_{3},{\tau }_{4} \right\} \end{eqnarray*}

(19) \begin{eqnarray*}{K}_{RAAPIV16}& = \left\{ {\tau }_{1},{\tau }_{2},{\tau }_{3},...,{\tau }_{16} \right\} \end{eqnarray*}

(20) \begin{eqnarray*}{K}_{RAAPIV64}& = \left\{ {\tau }_{1},{\tau }_{2},{\tau }_{3},...,{\tau }_{64} \right\} .\end{eqnarray*}

#### Feature vector formulation

In the concluding step of extracting features, a single feature vector was created and fed into the prediction model. Therefore, the following steps were taken in developing the final feature set: (1) Statistical moments were computed initially for PRIM and RPRIM for feature dimensionality reduction. (2) The resultant features were then assimilated into FV, AAPIV, and RAAPIV. Ultimately, a feature vector with 522 attributes was obtained. Each feature vector represents a single sample within the dataset. For binary classification, the positive samples were designated as “1”, and the negative samples were designated as “0”.

#### Feature scaling technique

A standard scalar technique (https://scikit-learn.org/stable/modules/generated/sklearn.preprocessing.StandardScaler.html) within the Python framework was used in this research study to standardise feature values obtained through the methods mentioned above. It is a common method to preprocess data before putting it into a computational model.

### Prediction models incorporation

Transforming raw biological sequences into discrete quantifiable vectors is a challenging task in artificial intelligence. The vectors serve as input to the machine learning algorithms such as random forest (RF), support vector machine (SVM), gradient boost (GB), *etc.* In this section, the development and training of prediction models are discussed in detail.

#### Random forest (RF)

A RF is an ensemble technique that combines various decision trees to get a more appropriate and accurate prediction result. Many decision trees participated in the classification, but, the majority voting by these decision trees won. The margin function $mg \left( X,Y \right) $ in Eq. (21) describes the ensemble of classifiers with the training set drawn at random from the distribution of the random vector *Y*, *X*.

(21) \begin{eqnarray*}mg \left( X,Y \right) =a{v}_{k}I \left( {h}_{k} \left( X \right) =Y \right) -ma{x}_{j\not = y}a{v}_{k}I \left( {h}_{k} \left( X \right) =j \right) .\end{eqnarray*}

Wenric & Shemirani (2018) utilised RF classifiers to rank genes by their expression values with the RNA-sequence sample set. This research implemented the algorithm using scikit-learn (version 0.24.2; https://scikit-learn.org/stable/). Optimal results were achieved by tuning hyperparameters such as maximum depth, maximum features, minimum samples, and the number of estimators. Nevertheless, tuning hyperparameters had a profound effect on the performance of the RF-based model (Probst, Wright & Boulesteix, 2019). In the current study, the *max_depth* was set to 100. Similarly, *max_features* was configured to Auto and *min_sample_leaf*, defining the number of samples required to be a leaf node, was set to 6. Following numerous experiments, the subsequent parameters for model training were determined, as shown in Table 1.

#### Support Vector Machine (SVM)

In supervised machine learning approaches, SVM is used for classification, regression, and outlier identification. In bioinformatics, SVM is well applied to the prediction problems of proteins and DNA/RNA sequences as well (Han, Wang & Zhou, 2019; Manavalan et al., 2019; Meng et al., 2019). Researchers (Feng et al., 2019; Xu et al., 2019) utilised SVM for the classification of D sites and non-D sites. The SVM-based model was deployed in the current study using the Python Scikit-learn library. Considering the hyper-parameters optimization through experiments, the following parameters were tuned to get the best results, as shown in Table 2.

#### K Nearest Neighbor (KNN)

KNN is a supervised machine learning method that uses training data to perform classification. It forecasts the values of new outcomes based on the closely matched training data points. Dongardive and Abraham (Dongardive & Abraham) conducted experiments using KNN with different neighbour values. They achieved the highest accuracy of 84%, with a neighbour value of 15 on the dataset containing 717 protein sequences. For experimental purposes, the research also employed the KNN model with the neighbour value (K) set to 3.

#### Artificial Neural Network (ANN)

A network of artificial neurons, often referred to as nodes, is what constitutes an ANN. These nodes are brought together to form a network that performs functions analogous to those of a biological neuron found in the brain. These nodes form different layers. There can be various hidden layers, with input and output layers. Each input signal is routed into a single input layer neuron before being passed on to the hidden layer. The final level of processing is completed by the output layer, which sends out output signals. ANN models have been extensively used in many research areas especially computational biology (Hussain et al., 2019a; Hussain et al., 2019b). In the present study, the ANN model was trained by modifying parameters such as hidden layer sizes, activation, solver, alpha, and learning rate, as indicated in Table 3.

**Table 1:** RF model tuning parameters.

**Parameter**	**Value**
N_estimators	1,000
max_depth	100
Max_features	Auto
Min_samples_leaf	6
Min_samples_split	10

**Table 2:** Hyperparameter optimization of support vector machine.

**Parameter**	**Value**
C	5
Probability	True
Gamma	‘auto’
Kernel	‘rbf’
Random_state	‘None’

**Table 3:** ANN hyperparameters tuning.

**Parameter**	**Value**
Random_state	1
Activation	‘relu’
Solver	‘adam’
Learning rate	0.001
Hidden_layer_sizes	5,2
Alpha	0.0002

## Results and Discussion

This research study was carried out to predict D sites located in tRNA using samples from three species through popular machine learning algorithms. Prediction models were developed and trained using a benchmark dataset. Models were evaluated through well-known metrics used in many research studies. For example, the current research study used four metrics for the evaluation of prediction models, including sensitivity (*S*_*n*_), specificity (*S*_*p*_), accuracy (*Acc*), and Mathew’s correlation coefficient (*MCC*).

### Metrics formulation

Four different metrics were used to evaluate the computational models including *S*_*n*_, *S*_*p*_, *Acc*, and the *MCC* as expressed in Eq. (22). *N*^+^represents the true D sites, whereas *N*^−^represents the rogue D sites. Similarly, the symbol ${N}_{-}^{+}$ shows the number of modified sites that were true D sites but incorrectly predicted as rogue D sites. Similarly, ${N}_{+}^{-}$ represents the number of rogue D sites incorrectly predicted as true D sites. However, it is necessary to mention here that metrics in Eq. (22) are only valid for single-class systems. For multiple classes of systems that are more prominent in biomedicine (Cao et al., 2018; Qiu et al., 2016), system medicine (Cheng et al., 2017), and system biology (Jain & Kihara, 2019), a completely different set of metrics are required as discussed in Chou (2013).

(22) \begin{eqnarray*} \left\{ \begin{array}{@{}l@{}} \displaystyle {S}_{n}=1- \frac{{N}_{-}^{+}}{{N}^{+}} 0\leq {S}_{n}\leq 1 \\ \displaystyle {S}_{p}=1- \frac{{N}_{+}^{-}}{{N}^{-}} 0\leq {S}_{p}\leq 1 \\ \displaystyle Acc=1- \frac{{N}_{-}^{+}+{N}_{+}^{-}}{{N}^{+}+{N}^{-}} 0\leq Acc\leq 1 \\ \displaystyle MCC= \frac{1- \left( \frac{{N}_{-}^{+}}{{N}^{+}} + \frac{{N}_{+}^{-}}{{N}^{-}} \right) }{\sqrt{ \left( 1+ \frac{{N}_{+}^{-}-{N}_{-}^{+}}{{N}^{+}} \right) \left( 1+ \frac{{N}_{-}^{\pm {N}_{+}^{-}}}{{N}^{-}} \right) }} -1\leq MCC\leq 1. \end{array} \right. \end{eqnarray*}

### Test methods

**Figure 4:**
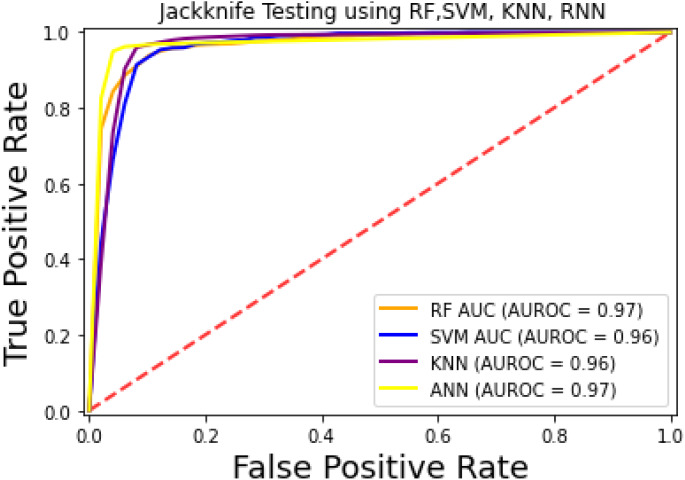
ROC-curve of jackknife test.

The prediction models used in this research study were evaluated through independent set tests, jackknife testing, and 10-fold cross-validation. The jackknife test usually imparts unique value to a similar dataset. Thus, in jackknife, the learning algorithm is applied once for each sample, using the selected sample as a single test set and all other samples in the dataset as the training set. The ANN revealed maximum *Acc*, *MCC*, and *S*_*n*_ scores. The jackknife test results are mentioned in Table 4. In Fig. 4, it is observed that the area under the curve of the RF-based predictor is at its maximum.

**Table 4:** Evaluation metrics result of jackknife test for RF, SVM, KNN, and ANN.

**Computational model**	***Acc***	***MCC***	***S*_*n*_**	***S*_*p*_**
RF	95%	0.90	0.92	0.97
SVM	94.6	0.89	0.97	0.92
KNN	92%	0.85	0.99	0.85
ANN	96.7%	0.93	0.98	0.95

**Figure 5:**
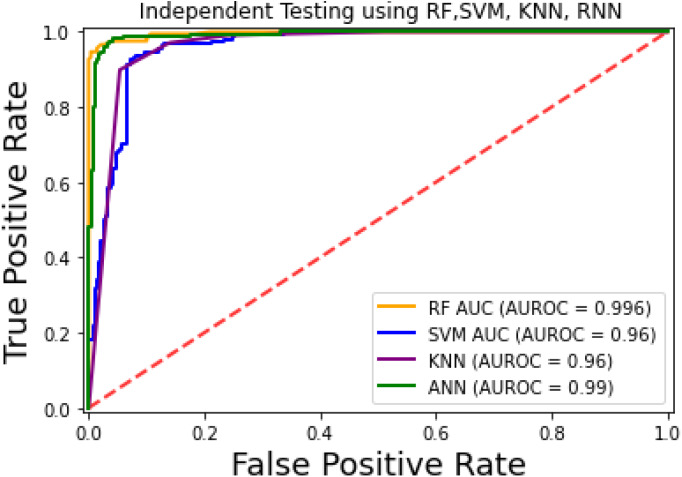
ROC-Curve of the independent set test.

**Figure 6:**
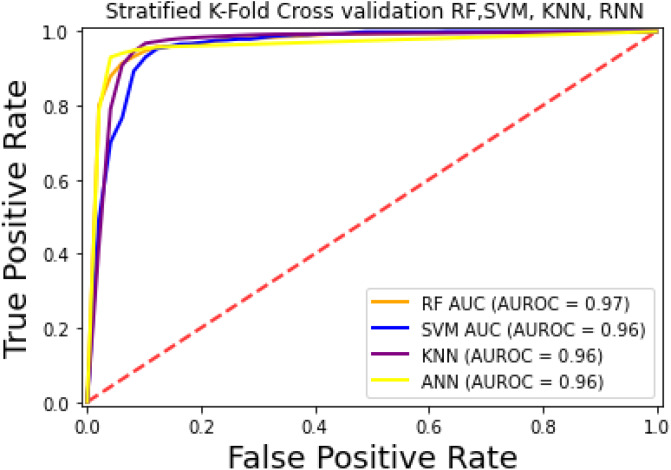
ROC-Curve of k-fold Cross-validation.

**Figure 7:**
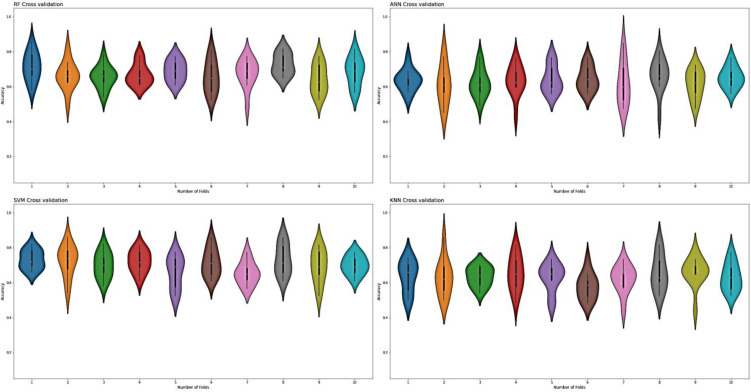
Violin charts RF, SVM, KNN and ANN cross validation.

**Figure 8:**
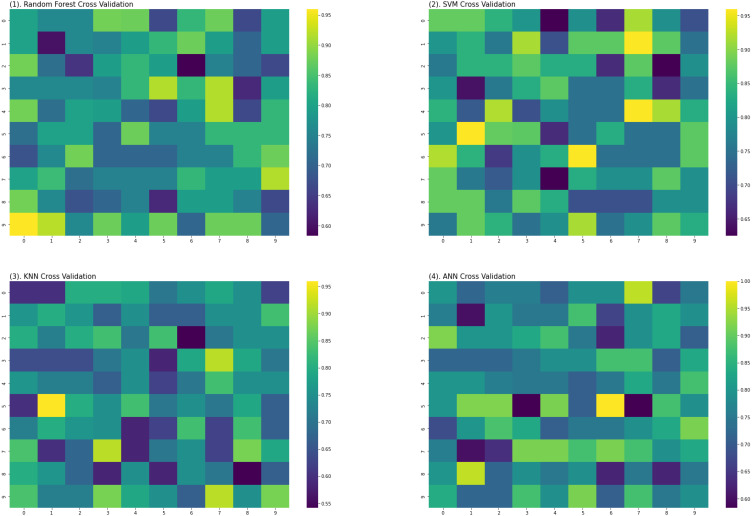
Heat maps of RF, SVM, KNN and ANN (cross validation results).

For the evaluation of models, an independent set test was used (Bui et al., 2016; Wójcikowski et al., 2019). The dataset was separated into two groups in this study, *i.e.,* the training dataset and the testing dataset. The dataset was split into an 80% training dataset and a 20% testing dataset for evaluation using the train-test split method of the python sci-kit learn library. It is essential to mention that the test samples were separate from the training samples during independent testing. The RF-based model revealed a maximum accuracy score of 96.9% in the independent set test. Similarly, the *S*_*n*_, *S*_*p*_, and *MCC* scores achieved by the RF-based model were the highest among the other three models as mentioned in Table 5. The results revealed that the RF-based model had shown a high AUC value compared to other models used in the study, as shown in Fig. 5. When the separate dataset is unavailable for validation, the cross-validation technique is adapted for model evaluation. The k parameter in K-fold cross-validation refers to the number of groups into which a sample of a given dataset should be divided. This test is widely used for evaluation due to limited data samples for validation. A 10-fold cross-validation was adopted in the current study. Through 10-fold cross-validation, RF divulged the maximum *Acc*, *MCC*, and *S*_*p*_ scores, among other models. as presented in Table 6. Cross-validation results have also been shown in the receiver operating characteristic (ROC) curve in Fig. 6, which depicts the area under the curve (AUC) of all the four prediction models used in this research. Violin plots and heat maps were used for visualizing cross-validation results. A violin plot uses density curves to represent numeric data distributions for one or more groups. For example, the median, interquartile range, and lower and upper adjacent values can be depicted through a white dot in the plot, a black bar in the center, and dark black lines stretched from the bar, respectively. Figure 7 shows violin plots representing accuracy values calculated in each fold for all prediction models. Moreover, heat maps can represent data graphically in the form of a matrix. Because they synthesize data and present it pictorially, heat maps provide an excellent visual summary of information. Its main advantage over other visualization tools is that it allows a large amount of information to be delivered fast. A heatmap is shown in Fig. 8, which depicts the cross-validation scores of all folds.

**Table 5:** Evaluation metrics result of the independent set test for RF, SVM, KNN, and ANN.

**Computational model**	***Acc***	***MCC***	***S*_*n*_**	***S*_*p*_**
RF	96.9%	0.93	0.98	0.97
SVM	91.5%	0.83	0.94	0.89
KNN	85%	0.74	0.97	0.75
ANN	96.3%	0.92	0.97	0.94

**Table 6:** Evaluation metrics result of 10-fold Cross-validation for RF, SVM, KNN, and ANN.

**Computational model**	***Acc***	***MCC***	***S*_*n*_**	***S*_*p*_**
RF	93.4%	0.86	0.92	0.94
SVM	93%	0.85	0.91	0.93
KNN	90%	0.81	0.97	0.85
ANN	92%	0.83	0.90	0.93

### Decision boundary visualization

**Figure 9:**
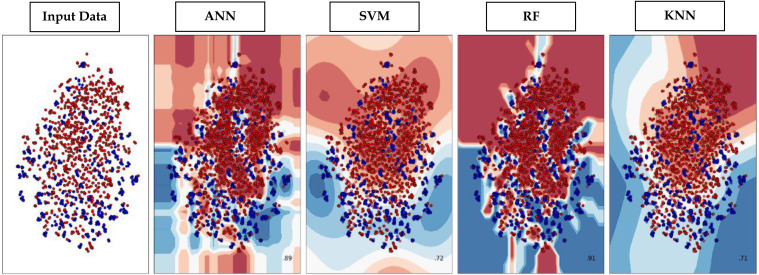
Decision Boundary plots of different classification algorithms used in this study.

Through supervised machine learning models, numerical prediction is sometimes not enough in many classification problems. It is critical to visualize the real decision boundary between the classes. Therefore, a decision surface was applied to the classification algorithms used in this work. A trained machine learning system predicts a coarse grid across the input feature space in this decision surface plot. First, the model was fitted onto the training dataset. Afterwards, the trained model was utilized to make predictions for a grid of values across the input domain. The contourf() function from matplotlib (https://matplotlib.org/3.5.0/api/_as_gen/matplotlib.pyplot.contourf.html) and scatterplot (https://seaborn.pydata.org/generated/seaborn.scatterplot.html) had been used for plotting. Figure 9 represents the decision surface plots of the classification algorithms employed in this study.

### Comparative analysis

For comparative analysis, two models, *i.e.,* iRNAD (Xu et al., 2019) and D-pred (Feng et al., 2019), were observed in this study. The benchmark dataset details of DHU-Pred, iRNAD, and D-pred have been shown in Table 7. After a thorough investigation of iRNAD and D-Pred, it was observed that only SVM was used for categorization. The current research study dealt with the development of four different prediction models, their evaluation through standard testing methods, and their performance comparison through standardised metrics. Moreover, the novel feature extraction method and the development of refined feature vectors helped achieve optimized results in predicting D sites. Therefore, the DHU-Pred outperformed the comparative models.

**Table 7:** Dataset information of DHU-Pred, IRNAD and D-Pred.

**Predictor**	**Database used for tRNA sequence retrieval**	**Species**	**Samples count**
DHU-Pred	RMBase	*Homosapiens, Mus musculus, & Saccharomyces Cerevisiae*	Positive = 1,035 Negative = 1,396
iRNAD	RMBase, Modomics	*Homosapiens, Mus musculus, Saccharomyces Cerevisiae, Escherichia coli, & Drosophila melanogaster*	Positive = 176 Negative = 374
D-Pred	RMBase	*Saccharomyces Cerevisiae*	Positive = 68 Negative = 68

Independent set testing was carried out on proposed and comparative models. It is essential to mention that the test samples differed from the training samples. Independent testing was carried out using 207 positive and 280 negative samples as mentioned in Table 8. However, k-fold cross-validation on the whole dataset was applied, in which the dataset was divided into 10 folds (for *k* = 10), such that in each of the 10 iterations, the model was trained using k-1 folds and then validated on the remaining fold. Therefore, the cross-validation approach adopted in this study was meticulous and different from that of independent set testing, where a specific separate sample was used for testing.

**Table 8:** Performance results of DHU-Pred, iRNAD, and D-Pred.

**Model**	***Test samples***	***Acc***	***S*_*n*_**	***S*_*p*_**	***MCC***	***F1-score***
DHU-Pred	Positive = 207 Negative = 280	96.9%	98%	99%	0.97	0.96
iRNAD (Xu et al., 2019)	Positive = 207 Negative = 280	91.6%	92.05%	98.13%	0.91	0.89
D-Pred (Feng et al., 2019)	Positive = 207 Negative = 280	85.2%	73.1%	97.2%	0.72	0.74

**Figure 10:**
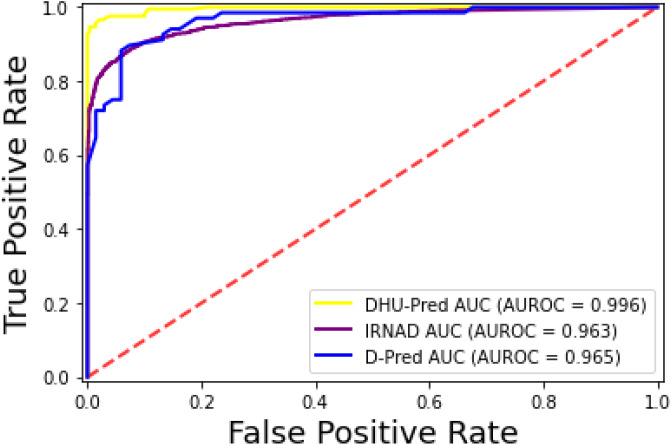
ROC-Curve of DHU-Pred, iRNAD and D-Pred.

The iRNAD and D-Pred revealed 91.6% and 85.2% accuracy, respectively, while DHU-Pred revealed a 96.9% accuracy score through independent testing, as in Table 8. The results in Table 8 show that the *Sn* and *Sp* scores achieved by iRNAD were 92.05% and 98.13%, while D-Pred revealed 73.1% and 97.2%, respectively. On the contrary, DHU-Pred revealed the *Sn* and *Sp* scores were 98% and 99%, respectively. The AUC-ROC graph in Fig. 10 also reveals that DHU-Pred outperformed both models, showing the high AUC value. This achievement was the comprehensive feature extraction method from the tRNA sequences.

Moreover, the inclusion of statistical moments into the obtained feature set helped build a more robust model for predicting D sites. The prediction of D sites is vital due to their role in the conformational flexibility of RNA and their significant presence in cancerous tissues. Therefore, the formulation of the benchmark dataset, the comprehensive method for feature generation and representation, the incorporation of different computational models, and evaluation through various testing methods helped us make a better model for D sites prediction than other available models. Therefore, based on the detailed experiments, it can be concluded that DHU-Pred, represents high accuracy, robustness, and expansibility for identifying the D modification sites.

## Webserver

The availability of a web server is essential because it provides a quick and easy means of computational analysis. Furthermore, the accessibility to such online tools helps researchers in any future developments. For this purpose, an online web server for the proposed model, DHU-Pred, was developed and is freely available at https://dhu-prediction-app.herokuapp.com/.

## Conclusion

Eukaryotes, bacteria, and even certain archaea all have high concentrations of D, a modified pyrimidine nucleoside. It aids nucleotide base conformational flexibility. Human pulmonary carcinogenesis is heavily influenced by this modification. In this research, computationally intelligent techniques were used to anticipate where D sites located in tRNA sequences. Features were computed for the stated goal using a convoluted approach based on statistical moments and position relative indices. The feature vectors were then incorporated into computational models for training. Cross-validation, jackknife testing, and independent set testing were used to assess these models. Using an independent set test, it was shown that the suggested RF-based model, DHU-Pred, revealed the highest results in all measures. DHU-Pred was compared extensively to popular academic models. Results from a comparison revealed that DHU-Pred performed far above the competition. As a result, the suggested model improved the identification capabilities of modified sites using the approaches described in the current study.

##  Supplemental Information

10.7717/peerj.14104/supp-1 Supplemental Information 1 Modified D-sites sequence (Positive samples) Positive fasta sequences having length 41 nucleotides each. The file can be best viewed through Bioedit software in windows OS.

10.7717/peerj.14104/supp-2 Supplemental Information 2 Non-modified D-sites sequence (Negative samples) Negative fasta sequences having length 41 nucleotides each. The file can be best viewed through Bioedit software in windows OS.

## Additional Information and Declarations

### DNA Deposition

The following information was supplied regarding the deposition of DNA sequences: The RNA sequence data used in this research study can be found in RMBase through the following URL: https://rna.sysu.edu.cn/rmbase/index.php. The raw data is also available in the Supplemental Files.

### Data Availability

The following information was supplied regarding data availability: The code and data are available at Github: https://github.com/taseersuleman/DHU-Prediction-app.
